# Time for the osteopathic profession to take the lead in musculoskeletal research

**DOI:** 10.1186/1750-4732-3-6

**Published:** 2009-07-22

**Authors:** John C Licciardone

**Affiliations:** 1 The Osteopathic Research Center, University of North Texas Health Science Center, Fort Worth, TX, USA

## Abstract

Musculoskeletal conditions, such as low back pain, are prevalent in the United States. These conditions exact an enormous toll on society, both in terms of their detrimental impact on quality of life and on the costs of treatment and lost productivity. Osteopathic physicians, as common providers of primary care services and spinal manipulation, are ideally positioned to lead future research efforts in this field. The emergence of data and standards relevant to osteopathic manipulative treatment outcomes, refinement of research methodologies to enhance evidence-based medicine, and investments in developing osteopathic research infrastructure are all critical elements in moving this field of research forward.

## The musculoskeletal research challenge

A few years ago, Deyo asked the rhetorical question, *"Where are the large trials involving dozens of practitioners and thousands of patients that are commonplace in cardiovascular disease and oncology and so scarce in musculoskeletal diseases?" *[1]. He concluded that, *"The nearly ubiquitous musculoskeletal conditions that 'merely' disable millions deserve equal attention and scientifically rigorous study." *[1]. Over 30 million ambulatory medical care visits annually are attributed to low back pain in the United States [2]. Correspondingly, the costs attributable to low back pain are enormous – exceeding $100 billion annually [3]. Conducting research to identify more effective ways of treating low back pain, particularly chronic low back pain, is a national imperative that will have a major impact on quality of life, health care expenditures, and work productivity.

## The osteopathic profession is ideally positioned to respond to this challenge

Osteopathic physicians in the United States manage a disproportionately large number of patients with low back pain [2]. In so doing, osteopathic physicians serve dual roles as providers of conventional medical treatment and spinal manipulation [4]. This dichotomy may explain why osteopathic physicians manage low back pain by prescribing drugs and physical therapy less frequently than allopathic physicians [2,5].

Data from the National Health Interview Survey (NHIS) in 2007 indicate that 14.3 million adults used complementary and alternative medicine (CAM) therapies for back pain within the past 12 months, making it by far the leading reason for CAM therapy use [6]. Neck pain, joint pain, and arthritis were the next three most common reasons for using CAM therapies. Crude frequency data from the 2007 NHIS also indicate that 36% of adults received spinal manipulation provided by an osteopathic physician or chiropractor in the past 12 months, and that 66% of these treatments were for back pain [7]. Many osteopathic manipulative treatment (OMT) techniques, such as high velocity-low amplitude thrusts, soft tissue techniques, and articulatory techniques, are comparable to those used by chiropractors and physical therapists [8]. Thus, OMT generally reflects the spinal manipulation provided by these other practitioners.

The volume of patients with low back pain managed by osteopathic primary care physicians and their dual roles as conventional and CAM practitioners suggest that the osteopathic profession is ideally positioned to respond to Deyo's challenge, particularly as it pertains to spinal manipulation for low back pain. The osteopathic profession should take the lead in providing the scientific evidence base to establish the safety, efficacy, and effectiveness of spinal manipulation in treating low back pain and other musculoskeletal conditions. This should involve a comprehensive approach, including basic science research, translational research, clinical trials, and comparative effectiveness studies.

## The evolution of clinical practice guidelines and scientific knowledge gaps relating to spinal manipulation for low back pain

Although spinal manipulation has been used in treating low back pain for over a century in the United States, the Agency for Health Care Policy and Research did not issue its seminal clinical practice guideline until 1994 [9]. It concluded that: (1) spinal manipulation can be helpful for patients with acute low back problems without radiculopathy when used within the first month of symptoms; and (2) in such patients having low back symptoms lasting more than one month, a trial of spinal manipulation is probably safe, but of unproven efficacy. Investigators in collaboration with the Cochrane Back Review Group subsequently performed a systematic review and meta-analysis and in 2003 concluded that spinal manipulation was neither more nor less efficacious than other standard (i.e., conventional) medical treatments for low back pain [10].

A review of nonpharmacologic therapies for acute and chronic low back in 2007 reported that the therapies with good evidence of moderate efficacy for subacute or chronic low back pain were cognitive behavioral therapy, exercise, spinal manipulation, and interdisciplinary rehabilitation, whereas only superficial heat was associated with good evidence of efficacy for acute low back pain [11]. The joint practice guideline from the American College of Physicians and the American Pain Society in 2007 recommended that clinicians consider adding spinal manipulation in the treatment of patients with low back pain who did not improve with self-care options [12]. It did not address the addition of spinal manipulation in the management of patients already receiving conventional medical treatment for low back pain.

Thus, spinal manipulation has been promoted as an *alternative *to conventional medical treatment for low back pain, but not as a *complement *to conventional medical treatment. The lack of guidelines on complementary spinal manipulation in the United States represents an important scientific knowledge gap because patients often seek conventional and CAM therapies concurrently.

In May 2009, the National Institute for Health and Clinical Excellence (NICE) in the United Kingdom issued its clinical guideline on the early management of persistent non-specific low back pain [13]. A goal of this guideline is to direct the management of low back pain in patients whose pain has lasted more than six weeks, with the objective of reducing disabling long-term back pain and thereby mitigating the personal, social, and economic impact of low back pain. The guideline recommended considering up to nine sessions of spinal manipulation over 12 weeks. It identified two other important research questions: (1) what is the effect of providing sequential or combination therapies when the initial monotherapy is inadequate?; and (2) what is the cost-effectiveness of providing such combination therapies in patients with persistent non-specific low back pain?

## The current state of research on osteopathic manipulative treatment for low back pain

Most studies of spinal manipulation for low back pain have focused on manipulation provided by chiropractors or physical therapists, not by osteopathic physicians or osteopaths. Although Goldstein challenged the osteopathic profession in 1997 to embrace evidence-based medicine [14], there have been no definitive mega-trials of OMT completed to date. However, in 2005, a systematic review and meta-analysis of clinical trials specifically addressed the use of OMT in treating low back pain [15]. The overall findings of this study indicated that subjects who received OMT experienced a greater reduction in low back pain compared with those subjects who did not receive OMT. Similar OMT benefits were demonstrated in a subgroup analysis comparing OMT with placebo or active treatments. A commentary on this study concluded that, *"...it gives clear, although still preliminary, evidence that OMT is much more than placebo," *and recommended that two sufficiently powered trials be conducted to better delineate the magnitude of OMT effects [16].

The OSTEOPAThic Health outcomes In Chronic low back pain (OSTEOPATHIC) Trial is a phase III clinical trial using a randomized, double-blind, placebo-controlled, 2 × 2 factorial design to study the efficacy of OMT [17]. Ultrasound physical therapy is the other factor studied in this trial. A total of 488 subjects (122 subjects allocated to each of the four treatment dyads) are to be recruited. At present, 770 participants have been screened, 349 subjects have been randomized, and 258 subjects have completed the trial.

Over the past few years, new data and standards have emerged to facilitate evidence-based medicine within the realm of OMT. A re-analysis of data originally collected in the North Texas Clinical Trial of OMT for chronic low back pain [18] was performed to assess the existence of the "OMT responder." Based on contemporaneous recommendations from the Cochrane Collaboration's Back Editorial Board regarding clinically relevant effects [10], and reported findings on the effects of placebo vs. no treatment in clinical trials involving pain outcomes [19], a 17-mm pain reduction on a 100-mm visual analogue scale was established as the criterion for successful response to OMT [20]. An international expert panel recently corroborated this approach by recommending minimally important changes on frequently used measures of pain and functional status for low back pain [21]. They considered 15 mm on a 100-mm visual analogue scale, five units on the Roland-Morris Disability Questionnaire, and 10 units on the Oswestry Disability Index to be minimally important absolute changes, and 30% improvement from baseline to be a minimally important relative change.

Having standards for minimally important changes enables the implementation of evidence-based medicine, including the determination of such measures as number needed to treat, number needed to harm, and likelihood of help vs. harm as they pertain to OMT [22]. Using such pain outcomes criteria, data from the North Texas Clinical Trial suggest that about 50% of patients with chronic low back pain may be successful responders to OMT within three months of initiating treatment. Prognostic factor analysis is another tool that can be used to identify significant predictors of successful response to OMT, and further enable the development of clinical decision rules [23]. The latter have the potential to inform not only future research design, but also clinical practice [24].

## Future directions for osteopathic research on musculoskeletal conditions

The spinal manipulation guidelines described above have been driven by "efficacy" studies (phase II or phase III clinical trials). Such studies generally include highly selected subjects treated under rigid experimental protocols. However, data are needed to corroborate the benefits of spinal manipulation in real-world settings under less-than-optimal conditions. Such "effectiveness" data, in some ways analogous to pharmaceutical post-marketing surveillance data (phase IV), are most efficiently collected in observational studies. Consequently, new paradigms are needed for extending research on OMT for musculoskeletal conditions to include thousands of patients. One such approach, for example, involves observation of osteopathic physicians providing OMT to their patients without the artificial constraints of a clinical trial protocol.

The Osteopathic Research Center (ORC) recently developed a framework for moving to this next step in musculoskeletal research, with a focus on chronic low back pain. The objectives of this ORC initiative include: (1) creating a Consortium for Collaborative Osteopathic Research Development (CONCORD); (2) training a cadre, initially of 24 clinical research fellows, with specific interests in OMT for chronic low back pain; and (3) developing a national practice-based research network (PBRN) using the clinical practices of these fellows. Ideally, the location and clinical practice characteristics of each fellow could be used to configure a geographically representative and population-based PBRN for research in this field. An inception cohort of 2,000 to 3,000 patients with non-specific chronic low back pain could be enrolled over two years, providing data on clinical and laboratory findings. The fellows' training would facilitate fidelity in documenting treatment interventions and measuring outcomes. This framework would provide a foundation for planning and implementing more rigorous studies as "spin-offs," including nested case-control studies, longitudinal studies, and clinical trials.

There is a great need for research involving the costs in providing OMT for musculoskeletal conditions [25]. Because providing OMT requires a substantial investment of physician time, often over many treatment sessions, simply documenting less concomitant drug or physical therapy use during the course of OMT may not be accepted as evidence of value by the purchasers of health care services. Cost-effectiveness analysis and cost-utility analysis are two established methods that should be used to study the cost implications of OMT.

Cost-utility analysis is particularly attractive for studying the effects of OMT provided as a complement to conventional medical treatment for low back pain. This approach measures the incremental costs of OMT needed to achieve an incremental improvement in health, often measured in quality-adjusted life years (QALYs). The costs of providing OMT in an ambulatory setting are often straightforward, and either can be directly measured by the health care provider or estimated using explanation of benefits statements or other third-party billing data. Recent advances in econometric modeling have led to the development of algorithms for converting common outcomes data, such as the Medical Outcomes Study SF-36, into QALYs [26]. Thus, these two elements of a cost-utility analysis (incremental OMT costs and incremental health benefits) can be used to compare conventional medical treatment + OMT with conventional medical treatment only in the management of low back pain.

## Conclusion

Musculoskeletal conditions, such as low back pain, are prevalent in the United States. These conditions exact an enormous toll on society, both in terms of their detrimental impact on quality of life and on the costs of treatment and lost productivity. Osteopathic physicians, as common providers of primary care services and spinal manipulation, are ideally positioned to lead future research efforts in this field. The emergence of data and standards relevant to OMT outcomes, refinement of research methodologies to enhance evidence-based medicine, and investments in developing osteopathic research infrastructure are all critical elements in moving this field of research forward.

## Competing interests

Dr. Licciardone is executive director of The Osteopathic Research Center.
